# Bibliographical Notices

**Published:** 1850-04

**Authors:** 


### BIBLIOGRAPHICAL NOTICES.

*New Publications.*—We have received a number of publications not included in the following list, which we have not found leisure to read, and of the merits of which we are not, therefore, prepared to speak. We mention this, that they, for whose attentions we are under obligations, may know the true reason of our apparent neglect, and not construe our omission to notice, into any want of appreciation of their kindness. On the publications noticed we should be glad to comment more at length, if time and space permitted. That we are unable to do full justice to our many bibliographical favors, cannot be more a subject of regret with our readers, than ourselves, however it may be with the authors whose works we receive in silence, or pass by with a cursory notice.

*Statistics of Cholera, with the Sanitary Measures adopted by the Board of Health, at Philadelphia, in the Summer of* 1849, *Chronologically analyzed.*

The statististical facts relating to the prevalence of Epidemic Cholera at Philadelphia are presented in this report, methodically arranged in a compendious form, convenient for reference.

*Report of the Proceedings of the Board of Health in relation to Cholera as it prevailed in New York in* 1849.

This contains a report of the Sanitary Committee to the Board of Health, embracing their proceedings, and in an appendix are presented the reports of Seth Geer, M. D., resident physician, Prof. Ellet, on the analysis of the atmosphere during the cholera, and of the medical officers individually, of four Cholera Hospitals opened in different parts of the city.

The statistics contained in this, as in the publication just noticed, are worthy of preservation, and contain important materials for a history of the epidemic, as it prevailed in the United States during the last summer.

---

*Report of the Pennsylvania Hospital for the Insane, for the year* 1849. By THOMAS S. KIRKBRIDE, M. D., Physician to the Institution.

The capacity of this institution, during the last year, has been increased by additional buildings. The total number in the hospital during the year was 408; the highest number at one time, 221. The institution embraces a farm and garden; a work-shop and mechanical department; a museum and reading room. Lectures, and other methods of instruction, and evening entertainments, enter into the system of treatment. The institution is one of the best regulated in the United States.

---

*Seventh Annual Report of the Managers of the State Lunatic Asylum, of New York, made to the Legislature, February* 4, 1850.

Ths whole number of patients treated at the asylum during the past year, is 867. Of these, 433 were males, and 424 females. Remaining at the close of the previous year, 495. Remaining at the close of the past year, 449. Admitted during the year, 352. Dysentery and small-pox prevailed to a considerable extent among the inmates during the past year. The medical report is made by Dr. George Cook, who was acting Superintent from the the time of the decease of the late Dr. Brigham, in September, to the appointment of the present incumbent, Dr. Benedict, in Dec. In addition to the usual statistics, the report contains an interesting table exhibiting the number of suicides committed in the different counties of this State, during the last five years, collected from fifty widely circulated Journals.

---

*Three Lectures preliminary to a Course on the Principles and Practice of Surgery.* By WILLIAM GIBSON, M. D. L. L. D., Prof. of Surgery in the University of Pennsylvania.

These lectures are devoted to notes of the medical men, hospitals, etc., of Germany, jotted by the author during an excursion abroad in 1847. Like a previous series of lectures, giving an account of his observations in England and on the continent of Europe, this collection makes not only a very readable publication, but contains much useful information. The distinguished author promises a new edition of his "rambles in Europe in 1849," with the addition of a full account of his pilgrimage in 1847.

*Lecture Introductory to the Course on Surgery, delivered at the Massachusetts Medical College, in Boston.* By HENRY J. BIGELOW, M. D., Prof. of Surgery.

This is emphatically *the* Introductory of the season. In sound philosophical thought, and classical elegance of style, it is admirable.

---

*West on the Diseases of Infancy and Childhood.* Philadelphia. Lea & Blanchard: 1850, 8 vo. pp. 451.

The lectures by Dr. WEST, originally printed in the London Medical Gazette, and afterward prepared by the author for separate publication, were first presented to the American reader in the Library department of the Medical News.

They contain the results of observations at a children's infirmary, for nine years, during which period nearly 14,000 children were brought under notice, accurate notes having been kept of 600 cases, and 180 dissections made in cases terminating fatally.

It will be perceived that the opportunity for the collection of materials for scientific investigation has been most ample. The author appears to have improved his advantages with judgment and skill, as well as industry and fidelity. We have perused a considerable portion of the work, and do not hesitate to pronounce it a valuable addition to the literature of infantile diseases.

For sale by Derby & Co.

---

*Posthumous work by Dr Forry.*—The late Dr. Forry, a short time before his decease, committed into the hands of Prof. C. A. Lee, a manuscript work on the subject of public hygiene, the laws of health, vital statistics, etc., in the preparation of which he had devoted much of the three last years of his life. The work is pronounced by those who have examined it, to evince great talent and erudition, calculated to diffuse much valuable information with reference to legislative and individual hygiene, and to confer honor on American medical literature. The late Albert Gallatin, who examined the work, deemed its publication highly important. The manuscript has remained for several years in the hands of Doctor Lee for want of a publisher. While our American publishers reproduce annually scores of foreign treatises, they are reluctant to risk the outlay required for the issue of this. At the last meeting of the New York State Society it was submitted, in connection with an able report by Dr. Lee on the subject of Hygiene, and its publication, by the Society, recommended. It was referred to the Committee on Publication, and it remains with that committee to decide whether it will be returned in manuscript to Dr. Lee, or presented to the American Medical public. What the decision of the committee will be, we have no means of knowing, but we do hope, on behalf of our national literature, that the work will appear under the auspices of our State Society. The State Legislature, as we learn, has passed an Act authorising the publication of the Transactions of the Society at the expense of the State. No difficulty therefore, in the way of funds, prevents its publication. This being the case, we trust that our State Society will not forego the honor of averting the disgrace of permitting this valuable contribution to our national Medical literature, to remain longer in manuscript.

---

*St. Louis Medical and Surgical Journal.*—We are glad to welcome the reappearance of this excellent periodical, which, phœnix-like, has arisen from the ashes of the great conflagration at St. Louis, the publication having been suspended since that epoch. We are glad also to see among the list of editors, the name of our friend, Prof. John B. Johnson. This accession completes an editorial quaternity. We trust our esteemed contemporary may never again pass through a fiery ordeal, either in a literal or metaphorical sense, but, if so, may it ever reappear as unscathed and fresh as it now does !

---

*Northern Lancet, aud Gazette of Legal Medicine; devoted to Medical Science, Literature, News, Criticism, Natural Philosophy, Botany, Natural History, Mineralogy, &c.* Edited by Francis J. Devignon, M. D., Ausable Forks; and Horace Nelson, M. D., Plattsburg, N. Y.

We neglected to notice in our last No. a new Medical periodical with the above title. It is a monthly publication of 32 large octavo pages, the subscription price being one dollar per annum. It is issued at Plattsburg, N. Y. The editors have commenced their labors with zeal and ability, which we hope will be properly encouraged and rewarded.

